# Hypolipidemic activity of *Dracocephalum kotschyi* involves FOXO1 mediated modulation of PPARγ expression in adipocytes

**DOI:** 10.1186/s12944-018-0893-3

**Published:** 2018-10-30

**Authors:** Shima Aslian, Razieh Yazdanparast

**Affiliations:** Institute of Biochemistry and Biophysics, University of Tehran, P.O. Box 13145-1384, Tehran, Iran

**Keywords:** Adipose, Diabetes, *Dracocephalum kotschyi*, Lipogenesis, PPARγ, SREBP-1

## Abstract

**Background:**

*Dracocephalum kotschyi,* as a wild-growing flowering plant (from Lamiaceae family), is locally prescribed for its various health-promoting properties including hypolipidemic and hypoglycemic effects. To evaluate the scientific basis of the traditional use of *Dracocephalum kotschyi* extract (DKE), we aimed to disclose its mode of action with main focus on white adipose tissue of diabetic rats.

**Methods:**

Streptozotocin-induced diabetic rats were exposed to different doses of DKE for 28 days followed by the determination of the sera biochemical factors. The oxidative stress status of the diabetic versus nondiabetic rats’ adipose tissue under the influence of DKE were also evaluated in terms of malondialdehyde (MDA) and some of antioxidant enzymes (superoxide dismutase, SOD, and catalase). Furthermore, we exposed 3T3-L1 cells to DKE and then evaluated both the extent of cells differentiation to adipocytes and measured the expression levels of some of the key signaling elements involved in adipogenesis and lipogenesis with main focus on PPARγ.

**Results:**

Our results indicated that DKE administration attenuated the levels of TG (triglycerides), TC (total cholesterol), LDL and blood glucose by 54, 40, 54 and 25%, respectively and enhanced the levels of HDL, catalase and SOD by 45, 74 and 56%, respectively. In addition to profound adipogenic and lipogenic effects on 3T3-L1 cells, DKE significantly enhanced p-AKT, p-FOXO1, PPARγ and SREBP-1 expressions while that of p-JNK was quenched parallel to effect of pioglitazone, an antidiabetic agent, used in our investigation as the positive control drug.

**Conclusions:**

Besides of confirming the hypolipidemic action of the plant, our results provided documents on at least one mode of action of DKE with profound effect on lipid metabolism in adipose tissue. Regarding our results, further investigation on DKE, as a new potential hypolipidemic alternative drug is warranted.

**Electronic supplementary material:**

The online version of this article (10.1186/s12944-018-0893-3) contains supplementary material, which is available to authorized users.

## Background

Adipose tissue abnormality is registered as one of the known diabetic complications linking diabetes to adipose tissue malfunction. It has recently being shown that insulin sensitizing action of a group of drugs known as thiazolidinediones could be restored following transplantation of wild-type adipose-tissue into diabetic mice [1]. Thiazolidinediones, as a group of antidiabetic agents, and similar to pioglitazone and rosiglitazone, act via activation of PPARγ (peroxisome proliferator-activated receptor gamma) which associates with other lipogenic factors like SREBP-1 (sterol regulatory element-binding protein 1) to upregulate adipocyte differentiation (adipogenesis) and lipid metabolism (lipogenesis) [2]. In that line, it has been shown that adipose specific PPARγ-knockout mice suffer from severe lipodystrophy and hyperlipidemia [3]. Thiazolidinediones, as PPARγ activators, induce adipose tissue remodeling where large adipocytes are replaced by small and more insulin sensitive cells [4].

On the other hand, partial protection against high fat diet-induced diabetes has been shown in FOXO1 (forkhead box O-1) haploinsufficient mice [5]. Based on the present literature data, FOXO1, as a suppressive transcription factor, attenuates PPARγ activity in adipocytes via binding and repressing the PPARγ promoter and/or via direct interaction with PPARγ protein [6]. Suppression of PPARγ activity is further intensified by JNK (c-Jun N-terminal kinase) activation via diabetes associated oxidative stress which facilitates nuclear localization and activation of FOXO1 in adipocytes [7].

Thus, modulation and mainly activation of PPARγ activity among diabetic patients might constitute a therapeutic strategy for diabetic patients. Antioxidants, with suppressive effects on JNK activity, would lead to FOXO1 activity suppression via AKT (also known as protein kinase B) activation with subsequent augmentation of PPARγ-associated lipogenesis and attenuation of blood glucose level. This mode of action might account for the hypoglycemic action of many herbal medicines rich in different groups of antioxidants.

In that line, we got interested to evaluate the probability of the aforementioned mechanism as the scientific basis for the traditional use of DKE as a hypoglycemic and hypolipidemic remedy in Iranian folk medicine with as yet unknown mode of action [8].

To document the lipogenic activity of DKE, in terms of PPARγ and FOXO1 activity modulation, we exposed a group of STZ (streptozotocin)-induced diabetic rats and 3T3-L1 (mouse embryonic fibroblast) cells to different doses of the extract for different time intervals as indicated in the text. Our data clearly confirmed the beneficial effects of DKE on PPARγ activation and FOXO1 deactivation in both the in vivo and the ex-vivo models and thus, scientifically approved the validity of the traditional use of DKE against diabetes and some of its complications.

## Materials and methods

### Aim and design of the study

For further investigations about hypolipidemic effects of *Dracocephalum kotschyi,* we prepared the plant’s hydroalcoholic extract and evaluated its antidiabetic and antioxidant properties. Regarding DKE’s definite hypolipidemic and moderate hypoglycemic effects, 3T3-L1 adipocytes were cultured in the presence of DKE and evaluated its adipogenic and/or lipogenic consequences. Furthermore, western blot analyses of total FOXO1, p-FOXO1, p-AKT, p-JNK, PPARγ and SREBP-1, as oxidative stress-related regulatory factors with established impact on lipid metabolism, were achieved to disclose the mode of DKE’s antidiabetic action.

### Materials

STZ, trichloroacetic acid and EDTA (ethylenediaminetetraacetic acid) were purchased from Sigma Chem. Co. (MO, USA); NADH (nicotinamide adenine dinucleotide), TBA (thiobarbituric acid) and NBT (nitroblue tetrazolium) were obtained from Merck Co. (Germany). Antibodies to p-AKT (Thr 308), p-FOXO1 (Ser 256) and FOXO1 were obtained from Cell Signaling Technology (MA, USA). Anti-PPARγ was purchased from Santa Cruz Biotechnology (Canada). Antibody to SREBP-1 was obtained from Abcam Co. (USA). Anti-tubulin and horseradish peroxidase conjugated secondary antibodies were supplied by Sigma Chem. Co. (Germany). Anti-JNK and anti-JNK1&2[pTpY ^183/185^] were obtained from Biosource (Nivelles, Belgium). All other chemicals were reagent grade and used without purification.

### Plant extract preparation

*Dracocephalum kotschyi* aerial parts were collected from Alborz province of Iran and identified by Dr. Zare (Tehran University Herbarium, deposition code: 47418). Dried plant powder was incubated in ethanol water solution (1:1, *v*/v) for an overnight at room temperature followed by centrifugation at 1000×g for 10 min. This process was repeated for two more times. The combined extracts were then subjected to vacuum evaporation at 70 °C to a final volume (ml) equivalent to plant powder weight (g). The concentrated extract was aliquoted and stored at − 20 °C.

### Cell culture

3T3-L1 preadipocytes were cultured in high glucose DMEM (Gibco. GI, USA) containing 10% fetal bovine serum and 1% penicillin/streptomycin at 37 °C and 5% CO2. Two days after confluency, the medium was replaced with DMI medium (high glucose DMEM containing 1 μM dexamethasone, 0.5 mM 3-isobutyl-1-methylxanthine, and 1.5 μg/ml insulin). After 48 h, the DMI medium was replaced by high glucose DMEM supplemented with 10 μg/ml insulin and 10% fetal bovine serum [9].

### Cell viability determination

The MTT [3-(4,5-dimethylthiazol-2-yl)-2,5-diphenyltetrazolium bromide] die was used to evaluate the cell viability. The cells (5 × 10^4^ /well) were seeded in 96-well plates and incubated with 90 μl complete DMEM. After 24 h of incubation, cells were treated with different concentrations (0–20 μl/ml) of DKE, for 24, 48 and 72 h and the cell viability was determined using 10 μl of MTT stock solution (5 mg/ml) for each well. Three hours after MTT addition, produced formazan crystals in each well were dissolved in 100 μl DMSO and measured at 570 nm.

### Determination of TG content in 3T3-L1 cells

After harvesting, 200 μl PBS was added to the cells and suspensions were sonicated in ice bath for 3 min followed by centrifugation at 10000 × g for 10 min at 4 °C [10]. Protein concentration was determined using Lowry’s method [11]. The TG content of each sample was determined using a relevant kit (Pars Azmun Co., Tehran, Iran) in accordance with manufacturer’s instructions.

### Oil red O staining and quantification

Oil Red O staining was performed as described previously [12]. In order to quantify, plates left to dry completely and 1500 μl of isopropanol was added to each well. Plates were incubated for 10 min at room temperature (wrapped with parafilm) and the concentration of dissolved Oil Red O was spectrophotometrically measured at 520 nm.

### Animal treatments

Seven days-acclimatized Wistar albino male rats, weighing between 220 and 250 g, were treated with a single intraperitoneal injection of STZ (45 mg/kg body weight, dissolved in sodium citrate buffer, 0.1 M, pH 4.5) and six rats (normal control group) were treated with the same volume of the vehicle. Three days after injection, the induction of diabetes was confirmed based on blood glucose level (above 250 mg/dl) and rat groups (*n* = 6) were subjected to daily gavage treatments for 28 consecutive days. Normal and diabetic control groups received 1 ml of distilled water. DKE-treated groups received 1 ml of 1:4 and/or 1:2 extract:water dilutions. Positive control group received 1 ml of pioglitazone (in distilled water).

### Biochemical analyses

Immediately after collection, blood samples were placed on ice and allowed to clot, centrifuged at 3500 × g for 20 min (at 4 °C) and serum aliquots were stored at − 70 °C. Serum levels of glucose, TC, TG, LDL and HDL were measured using relevant kits (Pars Azmun Co., Tehran, Iran) in accordance with the manufacturer’s instructions.

### Antioxidant enzyme assays and determination of lipid peroxidation

To obtain tissue homogenates [13] epididymal adipose tissues were quickly excised from sacrificed rats, washed twice with cold PBS, frozen in liquid nitrogen, pulverized and homogenized in the solution (1:2 *w*/*v*) containing 154 mM KCl and 3 mM EDTA, pH 7.4. Each homogenate was sonicated for 30 s and defatted by centrifugation, all steps were performed at 4 °C. After protein determination of each sample, the activity of SOD (superoxide dismutase) was assayed based on inhibition of NBT reduction to formazan [14]. One unit of SOD activity was expressed as 50% inhibition of NBT reduction per minute per mg of protein. Catalase activity was determined by measuring the decomposition of H2O2 per minute per mg of protein [15]**.**

The level of MDA (malondialdehyde) was determined via spectrophotometric determination of the purple color of the reaction mixture. Briefly, 250 μl of trichloroacetic acid solution (10%, *w*/*v*) was added to 50 μl of the defatted tissue homogenate, placed in a boiling water bath for 15 min, cooled to room temperature and centrifuged at 1000×g for 10 min. Then 200 μl of each supernatant was added to 100 μl of TBA solution (0.67%, *w*/*v*), placed in a boiling water bath for 15 min and the absorbance was measured at 532 nm at room temperature [16].

### Western blot analyses

Frozen adipose tissues (100–120 mg) were separately pulverized in liquid nitrogen. To each sample 0.2 ml RIPA lysis buffer was added followed by 30 min incubation on ice. Each homogenate was centrifuged at 14000×g for 15 min at 4 °C, defatted and the protein concentration was determined. Equal protein amounts were mixed with the loading buffer, electrophoresed and transferred to a polyvinylidene difluoride membrane. Membranes were blocked by 5% nonfat blocking solution for 1 hour and were then incubated overnight with the relevant primary antibody at 4 °C using BSA antibody dilution buffer followed by a 2 hour incubation at room temperature with the secondary antibody dilutions. The protein bands were detected by an enhanced chemiluminescence detection system (Amersham-Pharmacia, Piscataway, NJ) according to the manufacturer’s instructions. The supernatants of the 3T3-L1 cells’ extracts were also subjected to the same evaluation.

### Light microscopy and determination of adipocyte area

Adipose tissue depots (100–150 mg) were covered with 10% formaldehyde in phosphate buffer saline, pH 7.4, for 48 h at 4 °C, dehydrated in ethanol, cleared in xylene and placed within paraffin. Three serial sections of each sample (3 μm thickness) were stained with hematoxylin-eosin followed by light microscopic imaging at × 200 magnification. In this study, the adipocyte area was determined using the free open access software ImageJ (http://rsbweb.nih.gov/ij/) and 300 cells were considered for each evaluation.

### Statistics

Data are presented as the mean ± SD (standard deviation) of at least three independent tests and different cell cultures were used for each independent experiment. Significant differences were assessed by independent student’s t-test.

## Results

### DKE’s adipogenic and lipogenic effects in 3T3-L1 cells

**Fig. 1:**
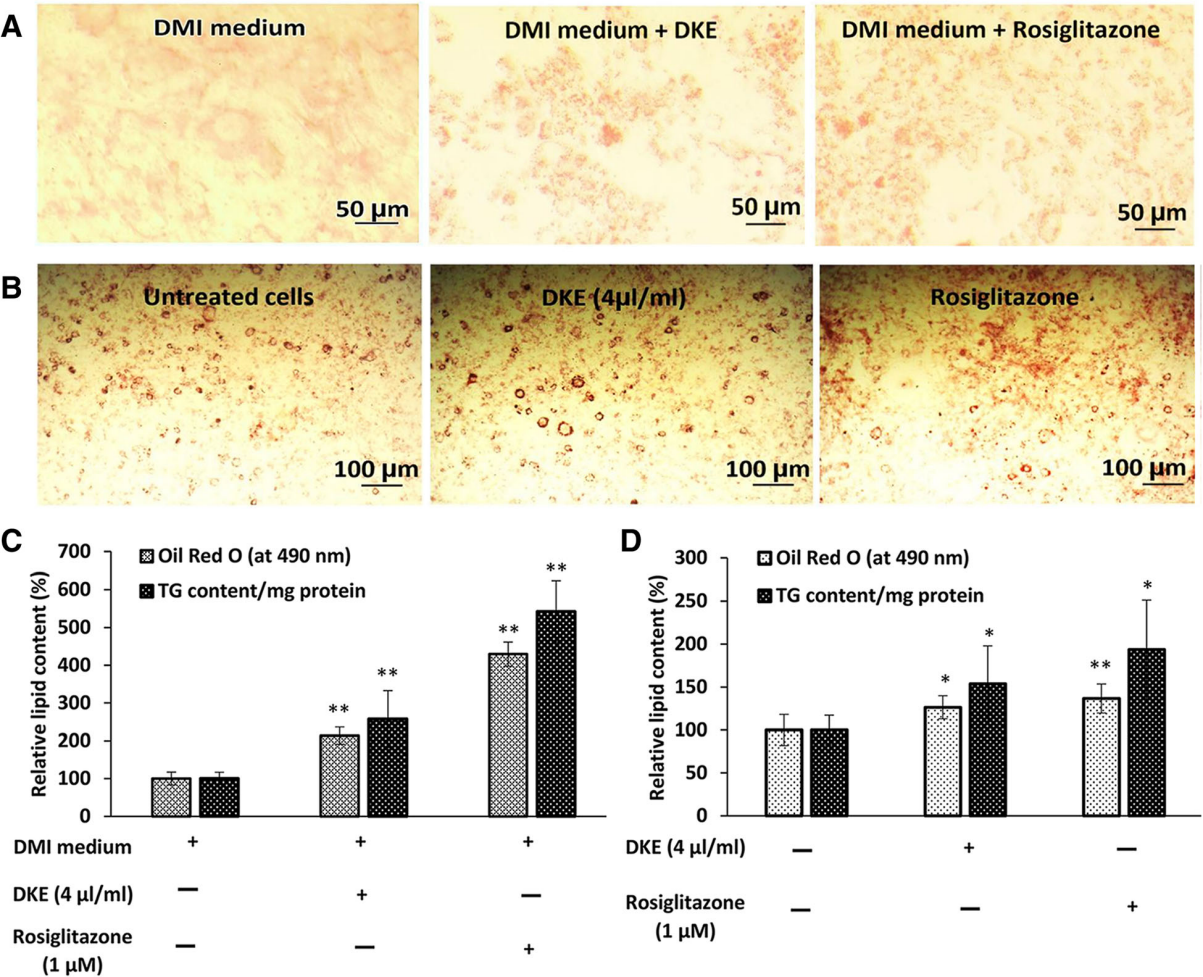
Evaluation of the adipogenic and lipogenic activities of *Dracocephalum kotschyi* extract (DKE) in 3T3-L1 cells. **a** and **c** cells were cultured for 48 h in DMI medium (high glucose DMEM containing 1 μM dexamethasone, 0.5 mM 3-isobutyl-1-methylxanthine, and 1.5 μg/ml insulin) in the presence or absence of DKE. Then, the DMI medium was replaced with high glucose DMEM containing 10 μg/ml insulin and the differentiation efficiency was evaluated after 6 more days by Oil Red O staining and triglycerides content was determined using the relevant kit. **b** and **d** to investigate DKE’s lipogenic effect, 3T3-L1 cells were differentiated to adipocytes. Eight days after differentiation, cells were treated with 4 μl/ml DKE and/or 1 μM rosiglitazone for 48 h and compared with untreated cells. The data represent a prototype outcome of the control, DKE- and rosiglitazone-treated cells (*n* = 3)

In most cases, the differentiation efficiency was very low using DMI medium. However, fully differentiated 3T3-L1 cells were obtained, in accordance to the literature data, using DMI medium supplemented with 1 μM rosiglitazone (Fig. 1a and c). [17]. Interestingly, the evaluation of TG content and Oil Red O cell staining indicated that DKE-supplemented DMI medium, without any effect on cell viability, increased the differentiation efficiency by about 2.57 folds compared to that of the control cells cultured in DMI medium without DKE supplementation (Fig. 1a and c).

To evaluate the lipogenic effect of DKE, 3T3-L1 differentiated adipocytes (8 days after the onset of differentiation) were treated for 48 h with 4 μl/ml DKE and/or 1 μM rosiglitazone. As presented in Fig. 1b and d, the triglycerides content of DKE-treated cells increased by almost 54% (*P* < 0.05, *n* = 3) relative to that of untreated control cells.

### PPARγ expression in DKE-treated 3T3-L1 cells

**Fig. 2:**
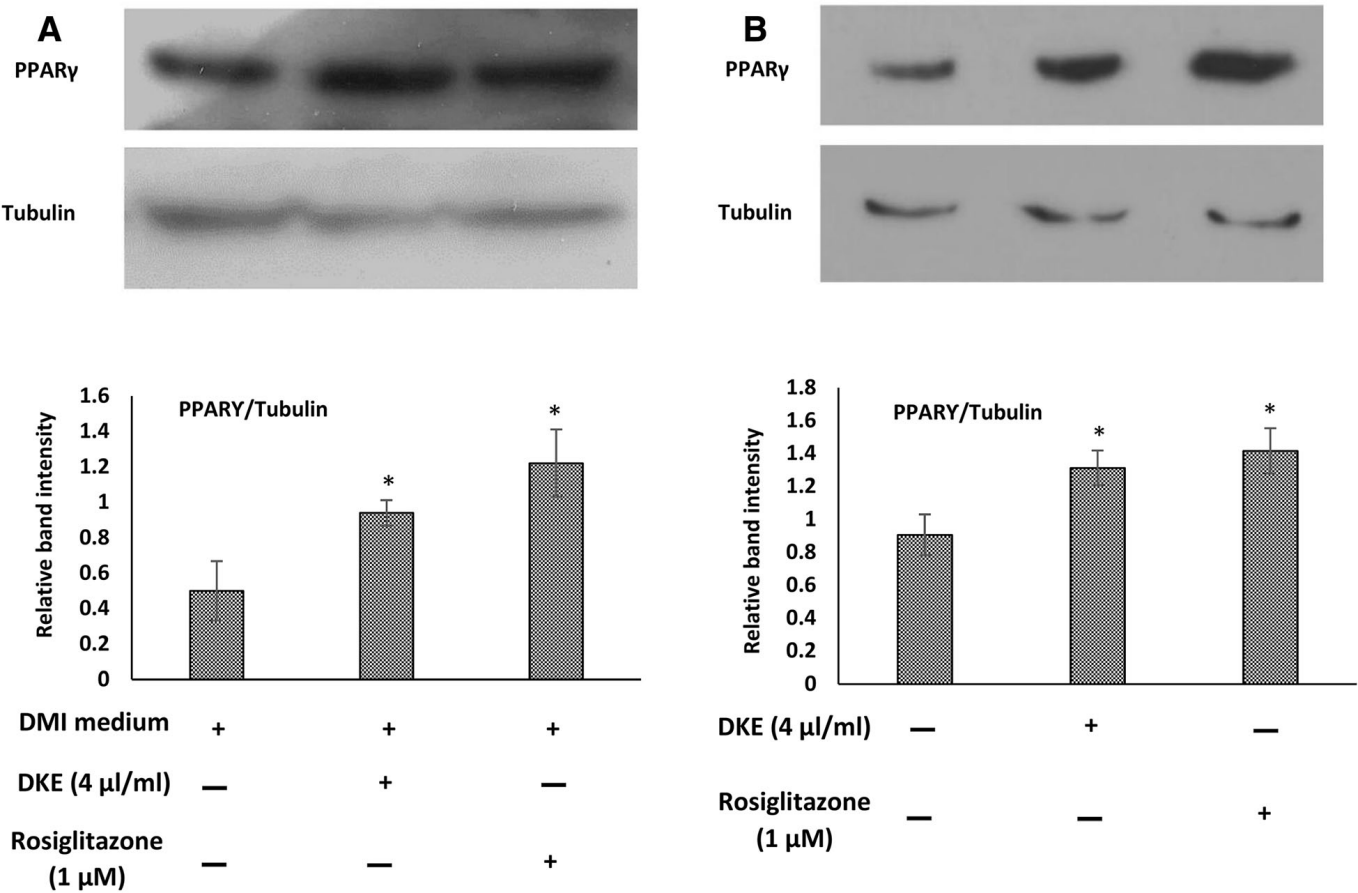
Western blot analyses of PPARγ in *Dracocephalum kotschyi* extract (DKE)-treated 3T3-L1 cells. **a** to observe DKE’s adipogenic effect, cells were differentiated using DMI medium (high glucose DMEM containing 1 μM dexamethasone, 0.5 mM 3-isobutyl-1-methylxanthine, and 1.5 μg/ml insulin). After 2 days, the DMI medium was replaced with high glucose DMEM containing 10 μg/ml insulin for another 6 days. The expression level of PPARγ was determined in cells treated with 4 μl/ml DKE and/or 1 μM rosiglitazone-supplemented DMI medium and compared to that of the control cells. **b** to investigate DKE’s lipogenic effect, differentiated cells were treated with 4 μl/ml DKE and/or 1 μM rosiglitazone for 48 h. The data represent means ± SD. *Significantly different from negative control (*P* < 0.05, *n* = 3)

As presented in Fig. 2a, the expression of PPARγ, the master regulator of adipogenesis and lipogenesis, increased by about 1.9 folds in 3T3-L1 cells exposed to DKE-supplemented DMI medium relative to cells exposed to DMI medium. Furthermore, following differentiation, the expression level of PPARγ was also increased by about 45% among DKE-treated adipocytes compared to that of untreated cells (Fig. 2b).

### In vivo investigations

As presented in Additional file 1, daily body weight measurement of the diabetic control group showed a body weight loss of around 25% after 5 weeks while that of DKE-treated group (0.5 ml/rat) was about 3%. Four weeks of treatment with DKE (0.5 ml/rat) decreased the level of water intake by 36% compared to untreated diabetic rats (*P* < 0.001, *n* = 6). Food intake evaluation of different experimental groups demonstrated an almost similar pattern to that of the water intake.

### Antidiabetic effects of DKE on STZ-induced diabetic rats

DKE-treatment of diabetic rats for 28 days (0.25 ml/rat) decreased the level of fasting blood glucose by 22% and the higher dose of DKE (0.5 ml/rat) resulted in a 25% decrease in fasting blood glucose level (Table 1). Moreover, 28 days of treatment with DKE (0.25 ml/rat) decreased the sera levels of TG, TC and LDL by 48, 31 and 43%, respectively, compared to the untreated diabetic group, whereas the dose of 0.5 ml/rat resulted in a decrease of 54, 40 and 54% in TG, TC and LDL content, respectively. The serum level of HDL was 45% higher in DKE-treated group (0.5 ml/rat) than that of the untreated diabetic group (Table 1).

**Table 1:** Antidiabetic effects of DKE

Groups	days	Glucose (mg/dl)	TG (mg/dl)	TC (mg/dl)	LDL (mg/dl)	HDL (mg/dl)
Normal control	0	131.25 ± 3.85*	61.73 ± 2.05*	66.45 ± 5.85*	18.01 ± 1.23*	34.02 ± 0.86*
	14	132.00 ± 2.20*	63.98 ± 3.52*	66.81 ± 3.44*	19.55 ± 1.78*	32.68 ± 1.44*
	28	127.00 ± 5.30*	62.33 ± 3.87*	67.88 ± 5.31*	20.41 ± 1.26*	35.04 ± 1.46*
Diabetic control	0	290.80 ± 18.20	120.44 ± 4.58	107.76 ± 5.67	56.72 ± 4.05	23.38 ± 0.77
	14	342.36 ± 3.96	127.36 ± 4.69	116.54 ± 6.04	65.22 ± 4.11	22.46 ± 1.61
	28	393.87 ± 7.97	143.36 ± 8.07	117.09 ± 1.46	76.90 ± 4.24	21.18 ± 1.82
DKE (0.25 ml/rat)	0	412.58 ± 17.82	110.35 ± 8.69	101.72 ± 6.22	66.11 ± 3.56	20.16 ± 1.30
	14	384.43 ± 11.07	93.33 ± 4.99*	99.53 ± 5.85	52.80 ± 1.56	23.57 ± 0.57
	28	321.07 ± 4.00*	74.15 ± 3.33*	80.74 ± 2.19*	43.75 ± 2.50*	24.34 ± 1.72
DKE (0.5 ml/rat)	0	379.43 ± 8.08	117.16 ± 8.22	106.77 ± 3.59	57.39 ± 1.85	22.16 ± 0.69
	14	365.22 ± 3.825	77.62 ± 3.08*	97.88 ± 2.37	44.17 ± 2.06*	26.64 ± 0.77
	28	284.80 ± 6.40*	66.33 ± 1.76*	69.89 ± 4.17*	35.26 ± 3.99*	30.74 ± 1.15*
Pioglitazone	0	342.73 ± 21.44	99.78 ± 4.70	116.91 ± 2.44	61.99 ± 3.57	23.23 ± 0.69
	14	255.89 ± 7.73*	72.66 ± 3.40*	92.14 ± 1.97*	41.26 ± 1.00*	29.13 ± 0.92
	28	178.67 ± 10.01*	63.63 ± 2.82*	72.38 ± 4.46*	24.51 ± 2.04*	33.25 ± 1.05*

Each value represents the mean ± SD (*n* = 6). *P < 0.05 statistically different compared to the diabetic control by Student’s *t*-test

### Antioxidant effects of DKE on adipose tissue

As demonstrated in Table 2, the adipose tissues of DKE-treated rats showed higher (*P* < 0.05, *n* = 6) catalase activities (54% and 74% at doses of 0.25 and 0.5 ml/rat, respectively) compared to the untreated diabetic group. Similarly, SOD activity was increased by %56 (*P* < 0.05, *n* = 6) in adipose tissue of DKE-treated rats (0.5 ml/rat). Measurement of MDA level in adipose tissue showed the potent ability of DKE to decrease (*P* < 0.05, *n* = 6) the oxidative damage (0.25 ml/rat, 43% and 0.5 ml/rat, 34%) compared to the untreated diabetic rats.

**Table 2:** DKE’s effect on antioxidative enzyme activities and MDA concentration in rats’ adipose tissues

Groups	Catalase (μmol/min. mg protein)	Superoxide dismutase (U/mg protein)	Malondialdehyde (nmol/mg protein)
Normal control	19.52 ± 0.54*	1.03 ± 0.06*	6.44 ± 0.36*
Diabetic control	10.24 ± 0.65	0.48 ± 0.02	14.73 ± 0.39
DKE (0.25 ml)	15.75 ± 1.01*	0.52 ± 0.12	8.44 ± 0.35*
DKE (0.5 ml)	17.79 ± 0.97*	0.75 ± 0.05*	9.73 ± 0.62*
Pioglitazone	18.99 ± 1.46*	0.81 ± 0.06*	9.07 ± 0.94*

The data represent the mean ± SD (*n* = 6). *P < 0.05 significantly different from the diabetic control group

### Increased adipocyte size in DKE-treated rats

**Fig. 3:**
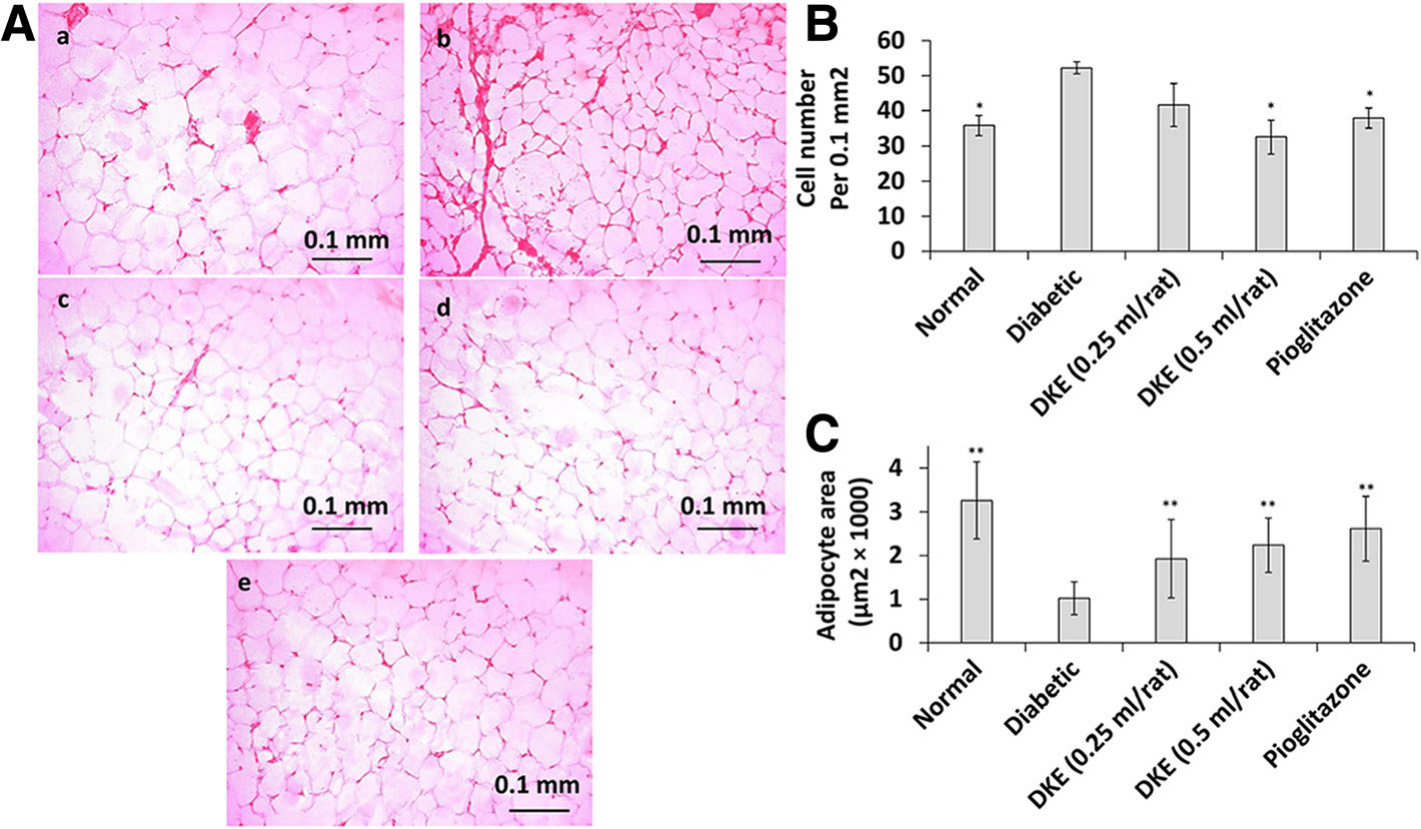
DKE-treated rats’ adipocyte size. **a** adipose sections were stained with hematoxylin-eosin and adipocytes were counted with light microscope at × 200 magnification. a: Normal rat; b: Diabetic control rat; c: DKE-treated rat (0.25 ml/rat); d: DKE-treated rat (0. 5 ml/rat); e: Pioglitazone-treated rat. **b** the number of adipocytes in 0.1 mm ^2^ of adipose sections. **c** adipocyte area of rat groups. The data represent means ±SD (*n* = 3). *P < 0.05 significantly different from diabetic control group. ** *P* < 0.01 significantly different from diabetic control group

To evaluate the DKE’s lipogenic effect, we determined the adipocyte number and respective area in rats’ epididymal white adipose tissue. The relevant data (Fig. 3b) demonstrated that the number of adipocytes (per 0.1 mm^2^) in adipose sections was decreased in DKE-treated rats (0.5 ml/rat) by about 38% compared to diabetic control group. In addition, DKE-treatment increased the adipocyte area by about 1.9 and 2.2 folds at 0.25 and 0.5 ml/rat (Fig. 3c), respectively.

### Decreased level of p-JNK in adipose tissue of DKE-treated rats

**Fig. 4:**
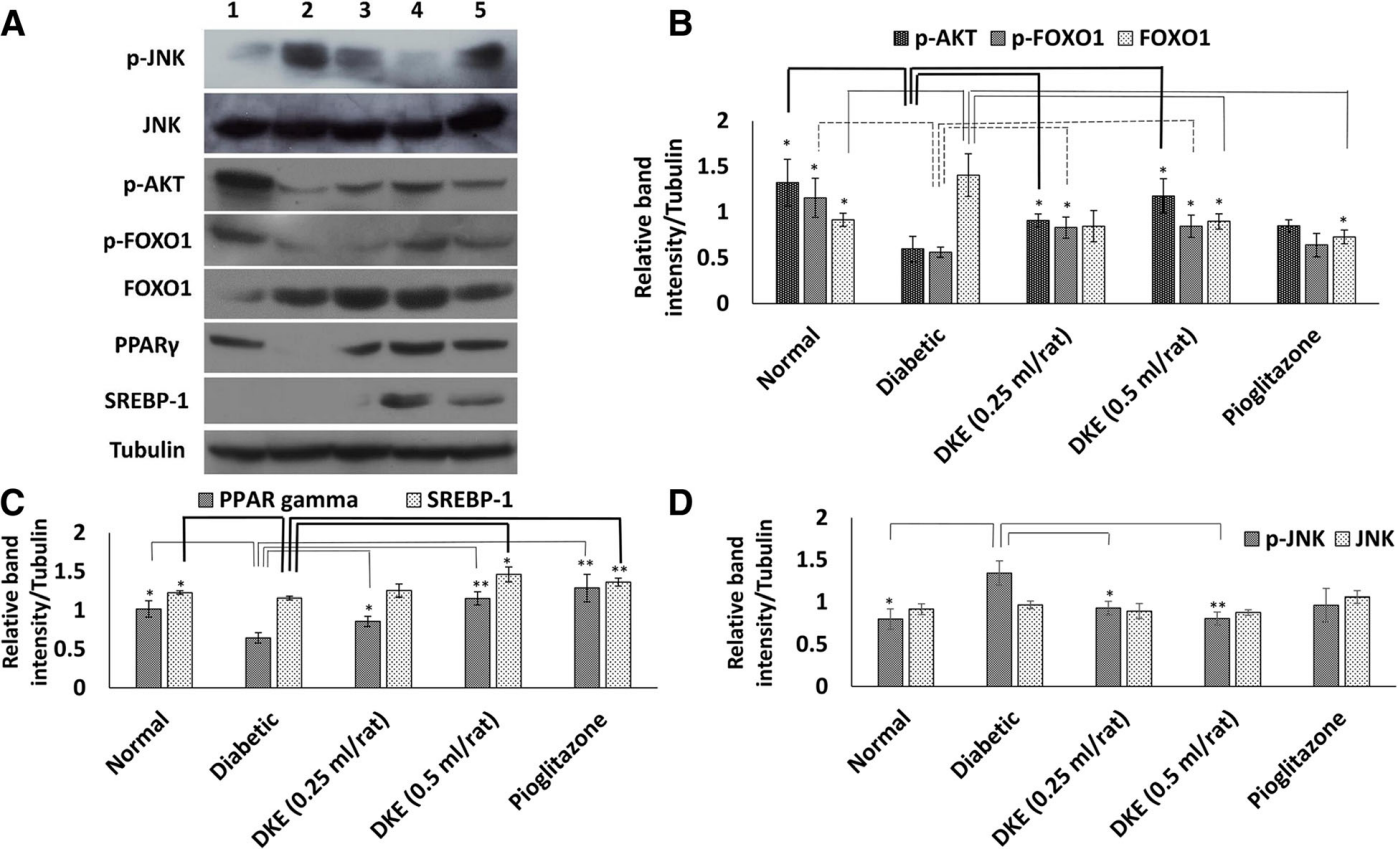
Western blot analyses of the adipose tissue of the streptozotocin-induced diabetic rats. **a** Diabetic rats were orally treated with *Dracocephalum kotschyi* extract (DKE) and/or pioglitazone (2 mg/rat). Adipose homogenates were analysed on a 10% SDS-polyacrylamide gel, transferred to PVDF membranes followed by incubation with the specific antibodies. Lanes 1 to 5: normal control, diabetic control, 0.25 ml/rat DKE-treated, 0.5 ml/rat DKE-treated and pioglitazone-treated group, respectively. **b**, **c** and **d** Relative band intensities after normalizing against corresponding tubulin bands. The data represent means ± SD. *P < 0.05 significantly different from diabetic control group (*n* = 3). **P < 0.01 significantly different from diabetic control group (*n* = 3)

The level of p-JNK, as evident from Fig. 4a and c, was increased by almost 68% in diabetic rats compared to the normal control group. However, DKE at both doses (0.25 and 0.5 ml/rat) decreased the phosphorylation level of JNK by about 31 and 40%, respectively, compared to the diabetic control group. The total JNK level remained unchanged in all experimental groups.

### Induction of the adipose tissue AKT level in STZ-induced diabetic rat

In adipocytes, the well worked out insulin-signaling pathway induces the activation of AKT that becomes evident by increased level of p-AKT [18]. As a consequence of STZ-toxicity in pancreatic beta cells and depletion of secreted insulin, the level of p-AKT decreased in adipose tissue of the diabetic control group by about 2.2 folds compared to normal rats (Fig. 4a and d). Our data indicated that DKE increased the p-AKT level by 52 and 97% at DKE doses of 0.25 and 0.5 ml/rat, respectively, compared to the control diabetic rats (Fig. 4a and d).

### DKE-mediated modulation of FOXO1 and p-FOXO1 levels

Induced kinase activity of AKT is anticipated to increase the phosphorylation level of one of its targets, FOXO1 [19]. Regarding this fact, the level of p-FOXO1 demonstrated a 48 and 51% increment at DKE doses of 0.25 and 0.5 ml/rat, respectively, compared to the diabetic control group (Fig. 4a and d). Despite these results, the total level of FOXO1 decreased by 36% at 0.5 ml/rat dose of DKE relative to the untreated diabetic rats (Fig. 4a and d).

### DKE-treatment induced the expressions of PPARγ and SREBP-1 in adipose tissue

Our western blot analyses indicated lowered levels of PPARγ expression among the diabetic group compared to the normal rats (Fig. 4a, column no. 2). However, after 28 days of DKE administration to the rats, the adipose tissue expression level of PPARγ increased by 33 and 79% at doses of 0.25 and 0.5 ml/ rat, respectively, compared to diabetic control group. Moreover, adipose tissue expression level of SREBP-1 increased by about 26% in DKE-treated group (0.5 ml/rat) compared to diabetic control rats (Fig. 4a, columns no. 3 and 4, and Fig. 4b).

## Discussion

Adipose tissue remodeling along with replacement of insulin-resistant adipocytes with younger and more insulin-sensitive cells is one of the therapeutic strategies to revile the diabetic complications. By this aim, thiazolidinediones have been used as PPARγ activators with adipogenic and lipogenic effects on adipose tissue. In this research, pioglitazone, a thiazolidinedione with established antidiabetic and antioxidant properties, was used as the positive control. Indeed our in vivo experiments reconfirmed pioglitazone’s antidiabetic and antioxidant effects along with modulated expressions of PPARγ, SREBP-1 and total FOXO1 in adipose tissue while, the levels of p-AKT and p-FOXO1 (involved in the insulin-signaling pathway) were not influenced among the positive control rats. Furthermore, our relevant results indicated a strong attenuation of the adipose tissue FOXO1 content among the DKE-treated rats. Consequently, the attenuated level of p-JNK was in line with higher phosphorylation level of AKT leading to down-regulation of FOXO1. Moreover, total FOXO1 expression was attenuated among the DKE-treated rats (0.5 ml/rat) compared to the diabetic control group. Actually, DKE has been shown to be rich in flavonoids as FOXO1 inhibitors, and previous investigations have demonstrated induced activities of PPARγ in several cell lines treated with these bioactive compounds [20, 21].

Based on our results, it could certainly be concluded that DKE possesses strong hypolipidemic activity with profound effects on the white adipose tissue. Although the fasting blood glucose level among the extract-treated rats was not restored to the normal level, there was a significant difference in the level of fasting blood glucose among the extract-treated and untreated diabetic rats.

Under insulin-signaling pathway, induction of lipogenesis occurs following activation of AKT and phosphorylation of FOXO1. Our data demonstrated an augmented insulin-signaling pathway in DKE-treated rats with a mechanism that had yet remained to be elucidated. Regarding the STZ-mode of action, it is believed that STZ, as a potent free radical precursor, enters the pancreatic beta cells through the glucose transporter, GLUT2, where it causes DNA alkylation-based cell death with subsequent attenuation of plasma insulin level. These conditions are, furthermore, associated with hyperglycemia, hyperlipidemia and oxidative stress not mentioning other detrimental side effects (for details, please refer to the reviews provided by Eleazu and Szkudelski) [22, 23]. It has been reported that pancreatic beta cells, relatively poor in oxidative stress defense systems, can be protected from diabetic oxidative stress through induction of endogenous antioxidant enzymes or exogenous antioxidant administrations. In fact, our laboratory has previously reported the effect of Teucrium polium extract (from Lamiaceae family) on the regeneration of the β-cell mass and insulin secretion in STZ-induced diabetic rats [24].

In addition to in vivo experiments, we also evaluated the adipogenic and lipogenic activities of DKE among 3T3-L1 cells which are wildly used as the ideal model system. Due to the differentiation capability of this cell line, it is expected some of the vital transcriptional factors involved in adipogenic and lipogenic processes such as PPARγ and SREBP-1 would undergo modulation. In fact our results confirmed this prediction among 3T3-L1 cells exposed to DKE and/or rosiglitazone, as the positive control. These observations were further supported by the increased adipocytes’ area of DKE-treated rats.

In summary, besides of disclosing the hypolipidemic action of DKE, our results provided documents on at least one mode of action of DKE and opened up the field for further evaluation of the plant extract, mainly structure and biological elucidation of DKE main active constituents.

## Additional file

Additional file 1: DKE’s effect on mean body weights, food and water intakes in STZ-induced diabetic rats. (PDF 154 kb)

## Abbreviations

AKT: protein kinase B
DKE: *Dracocephalum kotschyi* extract
EDTA: ethylenediaminetetraacetic acid
FOXO1: forkhead box O-1
JNK: c-Jun N-terminal kinase
MDA: malondialdehyde
NADH: nicotinamide adenine dinucleotide
NBT: nitroblue tetrazolium
PPARγ: peroxisome proliferator-activated receptor gamma
SOD: superoxide dismutase
STZ: streptozotocin
TBA: thiobarbituric acid
TC: total cholesterol
TG: triglycerides
