# Supplementary material for: Hypolipidemic activity of Dracocephalum kotschyi involves FOXO1 mediated modulation of PPARγ expression in adipocytes
Source: Lipids Health Dis. 2018 Oct 30;17:245. doi: 10.1186/s12944-018-0893-3 (PMC6208110; doi:10.1186/s12944-018-0893-3)
Supplement: Supplementary file 1 — DKE’s effect on mean body weights, food and water intakes in STZ-induced diabetic rats. (PDF 154 kb) [file 12944_2018_893_MOESM1_ESM.pdf]

Additional file 1

DKE's effect on mean body weights, food and water intakes in STZ-induced diabetic rats.

Group	Weeks	Body weight (g)	Food intake (g/rat.day)	Water intake (ml/rat.day)
Normal control	1	227.17±4.70	21.64±1.87	34.00±2.07
	2	230.67±4.81	20.86±1.28*	35.12±1.86*
	3	239.67±6.47*	22.45±1.13*	39.45±0.82*
	4	256.83±8.01*	24.24±0.70*	39.83±1.26*
	5	264.83±6.47*	25.93±0.68*	45.93±0.96*
Diabetic control	1	230.83±6.12	23.52±0.35	37.74±1.52
	2	224.17±5.84	27.67±0.32	64.36±5.78
	3	203.67±8.82	33.90±0.93	88.90±8.16
	4	186.00±7.09	40.69±0.54	95.93±4.72
	5	173.22±7.22	41.21±0.53	106.67±3.21
DKE (0.25 ml/rat)	1	238.67±8.31	26.62±3.87	36.81±0.89
	2	234.83±5.11	29.79±1.46	70.24±6.29
	3	229.67±6.01*	31.81±1.83	78.19±1.54
	4	228.33±5.57*	30.74±1.54*	87.64±2.26
	5	221.17±6.97*	29.69±0.92*	75.14±5.98*
DKE (0.5 ml/rat)	1	244.17±7.65	25.12±1.91	39.17±1.41
	2	237.00±5.83	28.07±0.91	69.36±5.89
	3	233.00±6.13*	30.12±1.24*	77.02±2.91
	4	234.50±5.79*	29.52±0.79*	71.31±2.80*
	5	237.83±5.37*	26.90±1.83*	68.43±3.62*
pioglitazone	1	230.67±8.30	22.26±1.70	40.38±1.10
	2	225.67±6.82	25.95±1.33	63.81±4.94
	3	228.83±5.76*	27.47±1.08*	69.21±4.02*
	4	219.67±6.57*	24.19±1.27*	59.00±3.66*
	5	221.83±4.52*	29.38±1.79*	58.90±1.23*

Data represent the mean ± SD (n = 6). * $P < 0.001$ significantly different from diabetic control group
